# COVID-19 SYMPTOMS AND RISK OF COGNITIVE FUNCTION IMPAIRMENT/DEMENTIA AMONG COMMUNITY-DWELLING OLDER ADULTS

**DOI:** 10.1093/geroni/igad104.2379

**Published:** 2023-12-21

**Authors:** Reza Amini

**Affiliations:** University of Michigan-Flint, Flint, Michigan, United States

## Abstract

Most studies investigating the correlation between cognitive function (CF) and COVID-19 are cross-sectional, with less than one-year intervals post-contracting COVID-19. Using three waves of the National Health and Aging Trends Study (NHATS), this study aims to investigate the correlation between COVID-19 and CF impairment/dementia among community-dwelling older adults. After excluding known cases of dementia, we used before (2019 N=3,292), during (2020 N = 2,756) and one year after COVID-19 pandemic (2021 N=2,379) data. The severity of the disease and COVID-19 preventing contact with family and friends (CFF) were self-reported (in 2020), executive function (EF) was measured by Clock Drawing Test (CDT), memory using delay 10-words recall test (DWRT), and NHATS dementia classification (in 2021). Multivariable ordered logistic regression results showed no significant correlation between dementia (i.e., normal, probable, and possible) and self-reported COVID-19 symptoms or preventing CFF. COVID-19 symptoms did not predict the risk of EF impairment. However, controlling for race/ethnicity interaction with CFF showed Blacks with limited CFF were 74% and 136% more likely to have dementia and EF impairment than their White counterparts, respectively. Experiencing COVID symptoms in 2020 increased the risk of delayed memory impairment in 2021 by 63%, while limited CFF increased this risk by 39%, which was 120% higher for Blacks than Whites. In conclusion, experiencing COVID-19 symptoms can affect delayed memory when this effect cannot be specified as dementia. The impact of social isolation caused by the COVID-19 pandemic on CF and increasing the risk of dementia is more harmful among Blacks than Whites.

